# Navitor™ in Evolut™: a case report illustrating a novel approach to redo-transcatheter aortic valve implantation

**DOI:** 10.1093/ehjcr/ytae577

**Published:** 2024-10-24

**Authors:** Sebastian Völz, Petur Petursson, Dan Ioanes, Truls Råmunddal, Oskar Angerås

**Affiliations:** Department of Cardiology, Sahlgrenska University Hospital, Blå Stråket 5, 41345 Gothenburg, Sweden; Department of Molecular and Clinical Medicine, Institute of Medicine, University of Gothenburg, Bruna Stråket 16, 41345 Gothenburg, Sweden; Department of Cardiology, Sahlgrenska University Hospital, Blå Stråket 5, 41345 Gothenburg, Sweden; Department of Molecular and Clinical Medicine, Institute of Medicine, University of Gothenburg, Bruna Stråket 16, 41345 Gothenburg, Sweden; Department of Cardiology, Sahlgrenska University Hospital, Blå Stråket 5, 41345 Gothenburg, Sweden; Department of Cardiology, Sahlgrenska University Hospital, Blå Stråket 5, 41345 Gothenburg, Sweden; Department of Molecular and Clinical Medicine, Institute of Medicine, University of Gothenburg, Bruna Stråket 16, 41345 Gothenburg, Sweden; Department of Cardiology, Sahlgrenska University Hospital, Blå Stråket 5, 41345 Gothenburg, Sweden; Department of Molecular and Clinical Medicine, Institute of Medicine, University of Gothenburg, Bruna Stråket 16, 41345 Gothenburg, Sweden

**Keywords:** Transcatheter aortic valve implantation, Coronary angiography, Cardiovascular disease, Transcatheter heart valve, Case report

## Abstract

**Background:**

Transcatheter aortic valve implantation (TAVI) is expanding to younger patients, and the management of valve failure is a growing clinical problem. The transcatheter heart valve (THV) of choice for redo-TAVI is a complex decision, and first reports have examined the use of the Sapien 3™ THV (Edwards Lifesciences; Irvine, CA, USA) in failed Evolut™ (Medtronic, Minneapolis, MN, USA) THV. However, several different technical approaches may be worth consideration, and the subject remains a matter of debate.

**Case summary:**

A 73-year-old female with previous coronary artery bypass grafting, percutaneous coronary intervention (PCI), and TAVI with a 26 mm Evolut™ R THV presented with signs of heart failure. The clinical investigation revealed a severe restenosis of the THV valve and a stenosis of the left coronary main stem. The patient was successfully treated with PCI of the left main and, during the same procedure, underwent implantation of a 25 mm Navitor™ THV (Abbott; Chicago, IL, USA).

**Discussion:**

This case presentation aims to illustrate the rationale of and necessary conditions for performing a TAVI-in-TAVI procedure with Navitor™ THV in a failed Evolut™ THV which, to our best knowledge, constitutes a novel approach to redo-TAVI.

**Learning points**

- Procedural planning using cardiac computed tomography is crucial in redo-transcatheter aortic valve implantation (TAVI) cases.
- Redo-TAVI with the Navitor transcatheter heart valve (THV) is a preferable option in a degenerated Evolut THV under certain conditions.
- Preserving coronary access is of great importance in redo-TAVI cases.

## Introduction

Transcatheter aortic valve implantation (TAVI) is expanding to younger patients, and the management of valve failure is a growing clinical problem. The transcatheter heart valve (THV) of choice for redo-TAVI is a complex decision, and first reports have examined the use of the Sapien 3™ THV (Edwards Lifesciences; Irvine, CA, USA) in failed Evolut™ (Medtronic, Minneapolis, MN, USA) THV.^1,2^ However, several different technical approaches may be worth consideration, and the subject remains a matter of debate. We present the implantation of a Navitor™ THV (Abbott; Chicago, IL, USA) in a failed Evolut R™ THV which, to our best knowledge, constitutes a novel approach to redo-TAVI resulting in single-digit transvalvular gradients in combination with preserved coronary access.

## Summary figure

**Figure:**
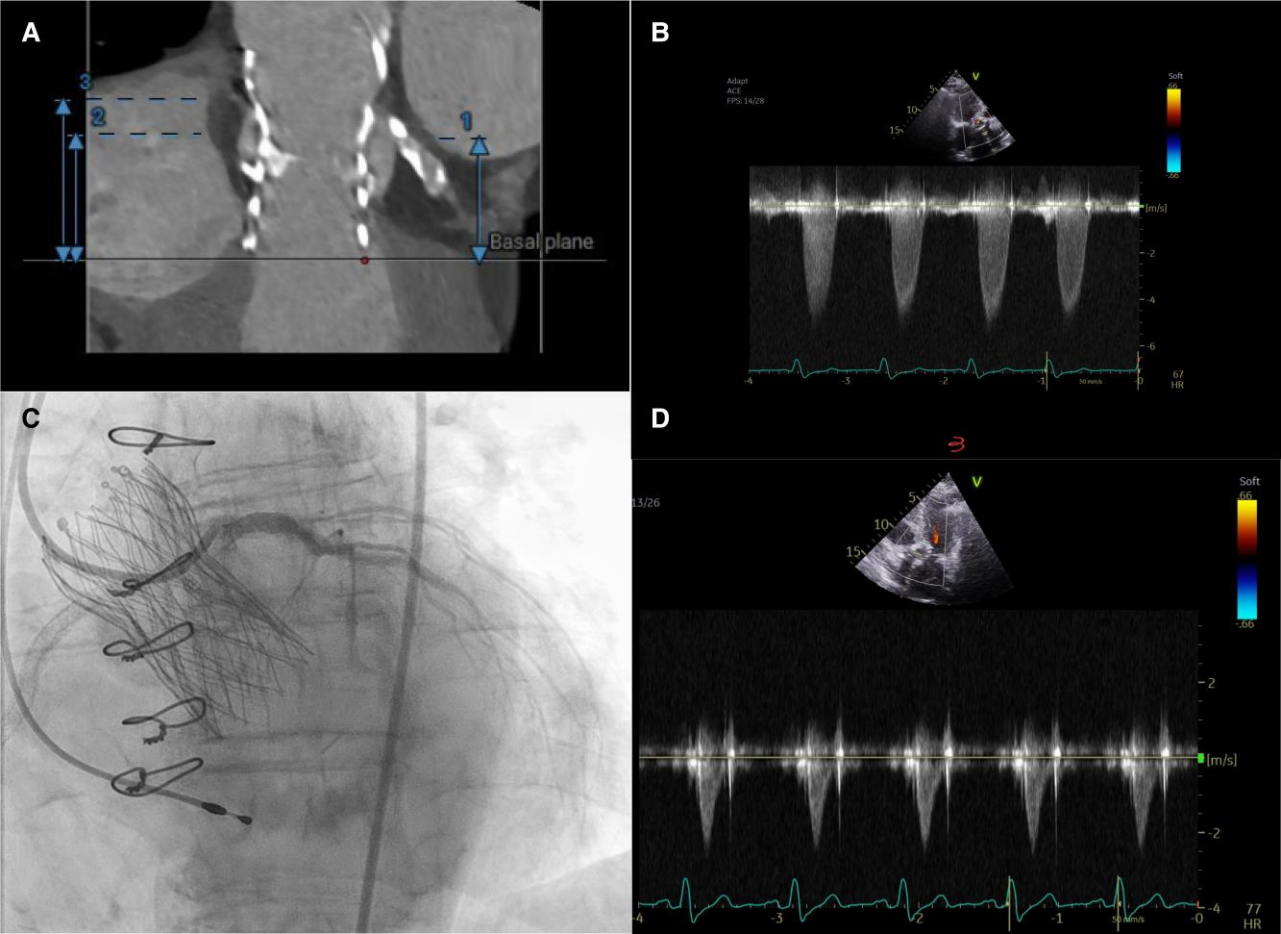
(*A*) Evolut R 26 mm TVH, height to (1) LCA ostium (26.3 mm), (2) RCA ostium (27.6 mm), and (3) ST-junction (35.1 mm). (*B*) Pre-procedural mean gradient of 59 mmHg over the Evolut R THV. (*C*) Reaccess of left main after PCI and implantation of Navitor 25 mm. (*D*) Post-procedural mean gradient of 9 mmHg.

## Case summary

**Figure 1:**
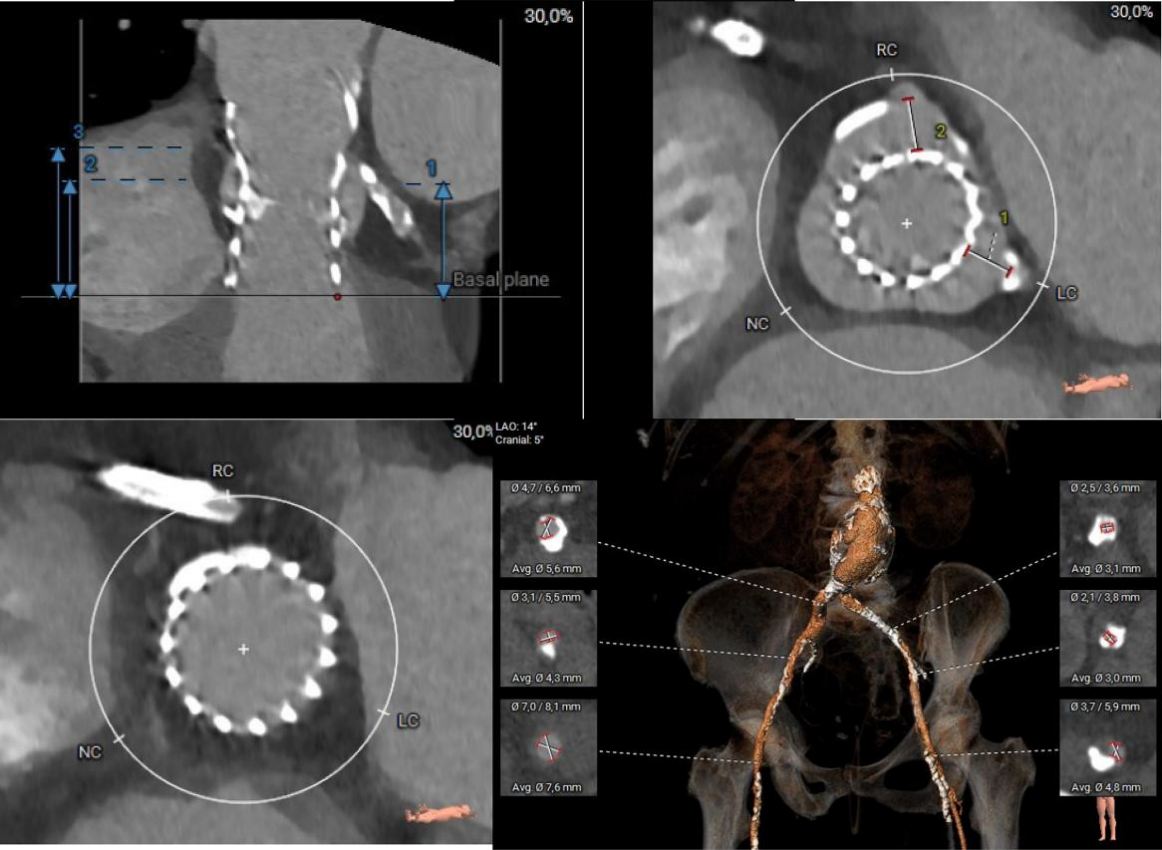
Pre-procedural CT scan with the index Evolut R 26 mm THV in relation to the aortic root and coronary ostia as well as access vessels. Upper left: depicts a stretched image of aortic root and index THV valve with measurement from the bottom of the THV stent frame to (1) LCA ostium (26.3 mm), (2) RCA ostium (27.6 mm), and (3) ST-junction (35.1 mm). Upper right: cross-section at the level of the offspring of the coronary arteries. VTC was measured to (1) 7.9 mm for the LCA and (2) 8.7 mm for the RCA, respectively. Bottom left: cross-section at the level of the STJ. Note that there is no space in between aorta and the expanded index valve. Bottom right: depicts the femoral and iliac arterial vessels. The minimal average diameter in the right vessel was 4.3 mm at the level of the internal iliac artery and in the left vessel 3.0 mm at the level of the common iliac artery. Note the aortic abdominal aneurysm.

A 73-year-old female patient was admitted for signs of heart failure. At admission, she had shortness of breath with a reduced SaO^2^ at 71%, elevated blood pressure of 170/90 mmHg, a systolic murmur on cardiac auscultation, and bilateral rales on lung auscultation. The patient had previously undergone coronary artery bypass grafting, consecutive percutaneous coronary intervention (PCI) as well as a transfemoral TAVI with a 26 mm EVOLUT R™ THV due to aortic stenosis in 2019. Echocardiographic assessment showed prosthesis failure with significant stenosis [mean pressure gradient (PG) 59 mmHg] and a severely hypertrophic left ventricle with preserved left ventricular ejection fraction. Coronary angiography displayed a patent left internal mammary artery-LAD graft but a borderline stenosis of the left main coronary artery (minimum lumen area 5.5 mm^2^). No evident cause for the early valve deterioration was identified. The predicted indexed effective orifice area of the initial THV device was 1.02 cm^2^/m^2^, and the height and weight of the patient were 1.68 m and 61 kg, respectively (body surface area 1.7 m^2^ and body mass index 21.6 kg/m^2^), hence patient prosthesis mismatch was of no concern.^3,4^ However, an elevated lipoprotein(a) (1.5 g/L) could potentially have contributed to the early development of the extensive calcified arterial and valve disease and hence the valve deterioration.^5^ A cardiac computed tomography (CCT) scan showed good commissural alignment of the index Evolut R™ THV. Coronary height was 26.3 mm for the left coronary artery (LCA) and 27.6 mm for the right coronary artery (RCA) as measured from the lowest point of the Evolut R™ stent frame. Valve-to-coronary distance (VTC) was 7.9 mm for the LCA and 8.7 mm for the RCA, respectively. At the sinotubular junction (STJ), the aortic diameter was equal to the diameter of the index valve. Both femoral arteries were severely calcified with a minimum lumen diameter of 4.3 mm in the right femoral artery (*Figure 1*). By transfemoral approach, the patient was first treated with a 4.0 × 12 mm Promus Elite drug-eluting stent (DES), post-dilated to 4.5 mm with a non-compliant balloon, in the left main and, during the same procedure, after the insertion of a GORE® Dryseal 18 F Introducer sheath, underwent implantation of a 25 mm Navitor™ THV including post-dilatation with a 24 mm TRUE™ balloon with good procedural result. The intervention was concluded with selective catheterization of the LCA (see Supplementary material online, *Videos 1*–*7*). Arterial closure was managed using a single Proglide™ in combination with an Angioseal 8F™ with no access complication. In-hospital echocardiography post-procedure showed low mean PG of 8 mmHg and no paravalvular leak. At 7-month follow-up, the echocardiography showed that the Navitor valve was well functioning with a mean PG of 6 mmHg and trace paravalvular leak. The patient was living independently with no signs of heart failure although she had suffered a non-disabling minor stroke not related to the valvular interventions.

### Rationale Navitor™ in Evolut™

We considered both redo-Evolut™ as well as the implantation of a balloon expandable valve (BEV) such as the Sapien 3™ or the Meril Myval™ (Meril Lifesciences, Vapi, India) THV. However, we decided on TAVI-in-TAVI with a Navitor™ 25 mm THV pursuing the following rationale: first, redo-Evolut™ was deemed unfavourable in terms of coronary access because of the valve’s small-cell stent frame, which was particularly relevant when considering the patient’s age and the newly implanted DES in the left main.^6^ In comparison with a redo-Evolut™, the low-profile stent frame of the Sapien 3™ THV was considered more favourable and an implantation would have been supported by the first *in vitro* and CCT studies simulating redo-procedures with Sapien 3™ in failed Evolut™ THV.^1,2,6^ However, with the Navitor™-valve, coronary access was not deemed to be at risk despite the unfavourable dimensions at the level of the STJ.^6^ Based on previously published bench testing, we could anticipate a maximum neoskirt height of ∼26.2 mm^2^ which in light of a STJ height of 35.1 mm and in spite of the notably small dimensions in STJ diameter would establish a low risk for sinus sequestration (*Figure 1*). Furthermore, the valve’s large cell design, and a VTC larger than 7 mm for both the LCA and RCA, was considered favourable in terms of post-procedure coronary perfusion and access. Of note, we concluded the procedure with uncomplicated LCA catheterization (see Supplementary material online, *Video 7*). Second, based on previous data, we presumed a favourable haemodynamic profile and a larger effective orifice area with a self-expanding as compared to the BEV Sapien 3™ however, although lacking randomized clinical data, compared to a supra-annular valve position, the intra-annular design of the Navitor™ THV does not seem to have a clinical significant negative impact on post-procedural gradients.^7,8^ The relief of PG to single-digit numbers post-procedure was considered a good haemodynamic outcome. Third, risk of leaflet overhang was eliminated with a self-expanding valve. Fourth, in order to keep the procedure transfemoral, despite the severely calcified iliofemoral vessels, the Navitor™ system was considered a better choice than the Edwards eSheath introducer which most probably would have demanded larger access vessel dimensions.^9,10^ Finally, in terms of life-time management, the implantation of an intra-annular valve such as the Navitor™ at this stage may be of advantage. By shifting the annular plane to a more inferior position, one consecutively lowers the level of the neoskirt that would arise in a potential future third procedure, thus maintaining the possibility to treat the patient percutaneously in case of repeat valve failure. In our opinion, the valve of choice in such setting should be a BEV. There are valuable electronic resources that provide guidance in terms of TAVI in TAVI.^11^ However, when consulting the mobile application, no data and thus no guidance were available on the combination Navitor™ in Evolut™.

## Conclusion

**Figure 2:**
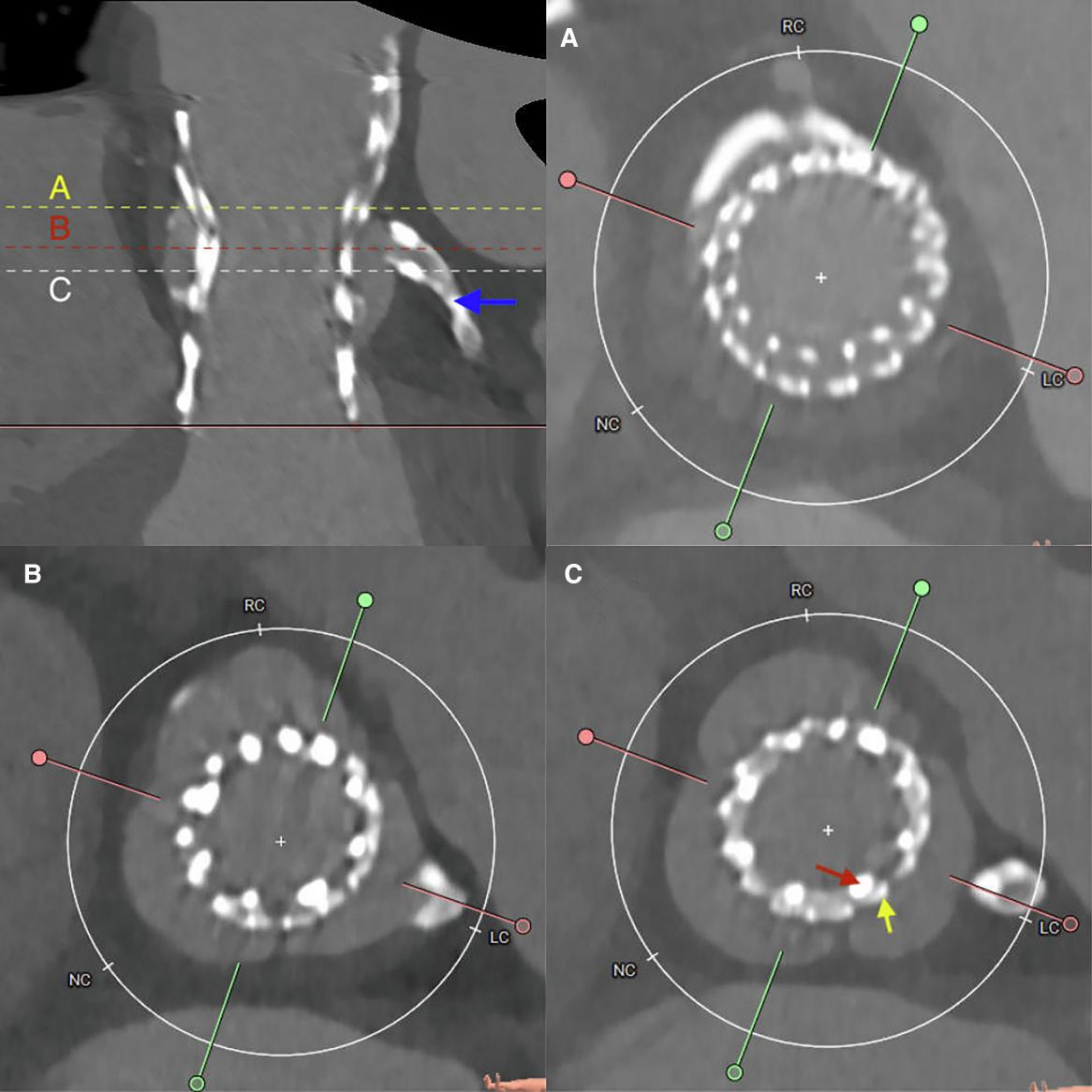
The image depicts aortic and valve dimensions after implantation of the Navitor™ THV. Upper left: depicts a stretched vessel projection of the aortic valve in the plane of the LCA (LCA marked with an arrow). (*A*) Cross-section at the level of the STJ. Note that there is no space in between aorta and the expanded index valve. (*B*) Cross-section at the level of the offspring of the left coronary artery. VTC was initially 7.9 mm for the LCA and 8.7 mm for the RCA, respectively, and remained unchanged after implantation of the Navitor™ THV. (*C*) Cross-section of the level of the highest point of the projected neoskirt, i.e. at 26.2 mm above the lowest point of the index Evolut. Note that the neoskirt and its actual height after implantation of the Navitor™ THV cannot be distinguished on the CCT scan. The red arrow depicts the stent frame of the implanted Navitor valve, the yellow arrow shows the stent frame of the index Evolut™ THV. STJ, sinotubular junction; VTC, valve-to-coronary distance; LCA, left coronary artery; RCA, right coronary artery; THV, transcatheter heart valve; CCT, cardiac computed tomography.

To our knowledge, this report constitutes the first description of TAVI-in-TAVI with Navitor™ in a failed Evolut™. The described valve intervention was feasible and may, from our point of view, constitute an option for the management of failure in Evolut™ valves. The post-procedural CT scan nicely depicts a well expanded Navitor™ THV valve within the Evolut™ THV with preserved prerequisites for coronary cannulation (*Figure 2*). Potential advantages over the use of BEV may include a favourable haemodynamic result, iliofemoral access despite hostile vessel anatomy, and the elimination of leaflet overhang. Also, the choice of an intra-annular device may be of advantage in a potential third intervention. However, it should be noted that the formation of a neoskirt is inevitable and sufficient coronary height above the index valve is necessary to maintain coronary perfusion and facilitate future coronary access. Tailor-made decision-making in these complex cases is key and may involve interventional cardiologist, imaging experts as well as cardiac surgeons. Bench testing and larger clinical datasets are required to further understand the implications of the described sequence of valves.

## Supplementary Material

ytae577_Supplementary_Data

## Data Availability

No new data were generated or analysed in support of this research.

## Lead author biography

Oskar Angerås is a senior consultant in interventional cardiology with a broad experience in coronary and valvular interventions at Sahlgrenska University Hospital. He has active research in the area of PCI and TAVI.

## Notes

**Consent:** The authors confirm that written consent for submission and publication of this case report including images and associated text has been obtained from the patient in line with COPE guidance.

## Notes

**Funding:** None.
